# Supplementary material for: Colistin plasma concentrations are not associated with better clinical outcomes in patients with pneumonia caused by extremely drug-resistant Pseudomonas aeruginosa
Source: Microbiol Spectr. 2023 Nov 9;11(6):e02967-23. doi: 10.1128/spectrum.02967-23 (PMC10715210; doi:10.1128/spectrum.02967-23)
Supplement: Tables S1-S4 and Figures S1-S3 — Antibiotic doses according to local guidelines, STROBE document, Css,avg related to estimated GFR, Multivariate analysis of mortality and clinical cure including Css as categorical variable and Kaplan-Meier also with Css,avg as categorical variable. Finally, a figure with Css avg cutoff point according to the maximally selected standardized log-rank statistic. [file spectrum.02967-23-s0001.docx]

**Supplementary material**

**Table S1.** Antibiotic doses according to local guidelines

| **Antibiotic** | **Dose** |
| --- | --- |
| Meropenem | 2 gr every 8 hours |
| Ceftazidime | 2 gr every 8 hours |
| Amikacin | 15 mg/kg/day (QD) + TDM |
| CMS:   - eGFR > 90 ml/min/m^2^ - eGFR 60-90 ml/min/m^2^ - eGFR 30-60 ml/min/m^2^ - eGFR 10-30 ml/min/m^2^ - eGFR < 10 ml/min/m^2^ | 3 million IU every 8 hours  2 million IU every 8 hours  1 million IU every 8 hours  1 million IU every 12 hours  1 million IU every 24 hours |

CMS: colistin methanesulfonate, eGFR: estimated Glomerular Filtration Rate (MDRD-4), IU: international units.

**Table S2. Checklist of items according to STROBE document.**

| **Title and abstract**   1. Indicate the study design with a commonly used term in the title or abstract 2. Provide an informative and balanced summary in the abstract of what was done and what was found | Study design specified in title and abstract  Balanced summary included in the abstract |
| --- | --- |
| **Background/rationale**  Explain the scientific background and rationale  for the investigation being reported | The scientific background and rationale are included in the Introduction |
| **Objectives**  State specific objectives, including any prespecified hypotheses | Pre-specified hypothesis and  objectives are stated in the Introduction |
| **Study design**  Present key elements of study design early in the paper | Study design described in the first part of Methods |
| **Setting**  Describe the setting, locations, and relevant dates, including periods of recruitment, exposure, follow-up, and data collection | Described in Methods |
| **Participants**   1. Give the eligibility criteria and the sources and methods of selection of participants. Describe methods of follow-up 2. For matched studies, give matching criteria and number of exposed and unexposed | Described in Methods  This is not a matched study |
| **Variables**  Clearly define all outcomes, exposures, predictors, potential confounders, and effect modifiers. Give diagnostic criteria, if applicable | Defined in Methods |
| **Data sources/ measurement**  For each variable of interest, give sources of data and details of methods of assessment (measurement). Describe comparability of assessment methods if there is more than one group | Specified in Methods. The same methods of data collection were used for groups. |
| **Bias**  Describe any efforts to address potential sources of bias | Selection bias: inclusion of consecutive cases.  Information bias: use of standard, well defined, easy to collect variables (piloted). Use of hard outcome variables. |
| **Study size**  Explain how the study size was arrived at | Study design does not require a sample size calculation |
| **Quantitative variables**  Explain how quantitative variables were handled in the analyses. If applicable, describe which groupings were chosen and why | Some quantitative variables were categorized according to statistical criteria (quartiles) to facilitate multivariate analyses |
| **Statistical methods**   1. Describe all statistical methods, including those used to control for confounding 2. Describe any methods used to examine subgroups and interactions 3. Explain how missing data were addressed 4. If applicable, explain how loss to follow-up was addressed 5. Describe any sensitivity analyses | Included in Methods  Included in Methods  Patients with missing data  were excluded  No patients were lost to follow-up  Included in Methods |
| **Participants**   1. Report numbers of individuals at each stage of study—eg numbers potentially eligible, examined for eligibility, confirmed eligible, included in the study, completing follow-up, and analysed 2. Give reasons for non-participation at each stage 3. Consider use of a flow diagram | Included in Results  Not applicable  Not applicable |
| **Descriptive data**   1. Give characteristics of study participants (eg demographic, clinical, social) and information on exposures and potential confounders 2. Indicate number of participants with missing data for each variable of interest 3. Summarise follow-up time (eg, average and total amount) | Table 1    There are no missing data  30-day information for all patients was available |
| **Outcome data**  Report numbers of outcome events or summary | Included in Results |
| **Main results**   1. Give unadjusted estimates and, if applicable, confounder-adjusted estimates and their precision (eg, 95% confidence interval). Make clear which confounders were adjusted for and why they were included 2. Report category boundaries when continuous variables were categorized 3. If relevant, consider translating estimates of relative risk into absolute risk for a meaningful time period | Specified in Results (Table 2)  Specified in methods. Only C_ss_ was categorized on the basis of quartiles. The C_ss_ cut-off was chosen on the basis of the maximally selected standardized log-rank statistic  Not applicable |
| **Other analyses**  Report other analyses done—eg analyses of subgroups and interactions, and sensitivity analyses | Specified in Methods and Results |
| **Key results**  Summarise key results with reference to study objectives | Specified in Abstract and Discussion |
| **Limitations**  Discuss limitations of the study, taking into account sources of potential bias or imprecision. Discuss both direction and magnitude of any potential bias | Included in Discussion |
| **Interpretation**  Give a cautious overall interpretation of results considering objectives, limitations, multiplicity of analyses, results from similar studies, and other relevant evidence | Included in Discussion |
| **Generalisability**  Discuss the generalisability (external validity) of the study results | Included in Discussion |
| **Funding**  Give the source of funding and the role of the funders for the present study and, if applicable, for the original study on which the present article is based | Included |

**Figure S1.** Dose-normalized colistin plasma concentration (C_ss, avg_) in relation to baseline estimated glomerular filtration rate (MDRD-4). Boxes represent the 25th, 50th and 75th percentiles, and the lower and upper whiskers the minimum and maximum values, respectively. Circles represent individual patient concentrations of colistin.

**Table S3. Multivariate analysis of mortality including C_ss, avg_ as a categorical variable (optimal, overexposure, underexposure)**

| **Variable** | **HR** | **P** | **[95% Conf. Interval]** |
| --- | --- | --- | --- |
| SOFA | 1.23 | 0.001 | 1.08-1.39 |
| C_ss, avg_ optimal*:   - underexposure - overexposure | 1.11  4.57 | 0.892  0.009 | 0.24-5.08  1.46-14.3 |
| Charlson comorbidity index | 1.05 | 0.489 | 0.92-1.20 |

C_ss,avg:_ colistin plasma concentrations at steady-state. HR: hazard ratio, SOFA: Sepsis-Related Organ Failure Assessment score.

*Reference category.

C_ss,avg_ optimal (0.625 to 1.25 mg/L), C_ss,avg_ underexposure (<0.625 mg/L), Css overexposure (C_ss,avg_ > 1.25 mg/L)

**Table S4: Multivariate analysis of clinical cure including C_ss, avg_ as a categorical variable (optimal, overexposure, underexposure)**

| **Variable** | **OR** | **P** | **[95% Conf. Interval]** |
| --- | --- | --- | --- |
| SOFA | 0.70 | 0.001 | 0.57-0.90 |
| C_ss,avg_ optimal*:   - underexposure - overexposure | 1.48  0.32 | 0.661  0.1 | 0.25-8.81  0.08-1.24 |
| Charlson comorbidity index | 0.80 | 0.093 | 0.62-1.03 |

C_ss,avg:_ colistin plasma concentrations at steady-state. OR: odds ratio, SOFA: Sepsis-Related Organ Failure Assessment score.

*Reference category.

C_ss,avg_ optimal (0.625 to 1.25 mg/L), C_ss,avg_ underexposure (<0.625 mg/L), Css overexposure (C_ss,avg_ > 1.25 mg/L)

**Figure S2. Cumulative Kaplan-Meier estimates of the overall probability of 30-day survival at day 30, stratified by three C_ss, avg_ groups (optimal, underexposure, overexposure)**

Log-rank test P = 0.0017

C_ss,avg:_ colistin plasma concentrations at steady-state

C_ss,avg_ optimal (0.625 to 1.25 mg/L), C_ss,avg_ underexposure (<0.625 mg/L), Css overexposure (C_ss,avg_ > 1.25 mg/L)

**Figure S3. Css, avg cutoff point according to the maximally selected standardized log-rank statistic**

C_ss,avg:_ colistin plasma concentrations at steady-state

**Cutoff point = 1.5 mg/L. P-value for the log-rank test = 0.0004513**
